# Can Quantitative CT Scan Analysis of Pulmonary Parenchymal and Vascular Characteristics Help to Differentiate Among Various Phenotypes of COPD Associated With Pulmonary Hypertension?

**DOI:** 10.1016/j.chpulm.2026.100244

**Published:** 2026-02-27

**Authors:** Ahmad Raza, Parth Rali, Mario-Tovar Naranjo, Shameek Gayen

**Affiliations:** a Department of Thoracic Medicine and Surgery, Temple University Hospital, Philadelphia, PA; b Department of Thoracic Medicine and Surgery, Lewis Katz School of Medicine, Temple University, Philadelphia, PA

**Keywords:** COPD-PH phenotypes, pulmonary hypertension, quantitative CT scan, COPD

## Abstract

**Background:**

Quantitative CT imaging has been applied to pulmonary vascular disease, but its role in characterizing phenotypes of COPD associated with pulmonary hypertension (COPD-PH) has not been evaluated systematically.

**Research Question:**

Can quantitative CT imaging reliably identify and differentiate major COPD-PH phenotypes?

**Study Design and Methods:**

We conducted a retrospective cohort study of patients with COPD-PH at Temple University Hospital from 2010 through 2022. Patients were categorized into 3 phenotypes: pulmonary vascular (pulmonary vascular resistence [PVR] > 5 Wood units [WU]; FEV_1_ > 50% predicted), pulmonary parenchymal (PVR < 5 WU; FEV_1_ < 50% predicted), and mixed (PVR > 5 WU; FEV_1_ < 50%). Quantitative CT imaging-derived vascular measures (proportion of blood volume in pulmonary vessels with cross-sectional area of < 5 mm^2^ [BV5] and proportion of blood volume in pulmonary vessels with cross-sectional area of < 10 mm^2^ [BV10]) and parenchymal measures (regional lung volume, total emphysema volume, emphysema percentage) were compared using analyses of variance.

**Results:**

Ninety patients met inclusion criteria. Mean PVR was 9.5 WU, 7.5 WU, and 2.8 WU in the vascular, mixed, and parenchymal phenotypes, respectively; corresponding mean FEV_1_ values were 67.4%, 35.8%, and 22.3%. Survival analysis demonstrated significantly higher mortality in the mixed phenotype (31%) vs the vascular (14.2%) and parenchymal (6.7%) groups (*P* = .04, log-rank test). Cox regression confirmed increased mortality risk in the mixed phenotype (OR, 2.12; 95% CI, 1.17-3.86; *P* = .001). Quantitative CT imaging-determined phenotypes showed similar patterns, with mortality of 75%, 25%, and 7.1% in the mixed, vascular, and parenchymal groups, respectively. The BV5 to total vessel volume ratio predicted elevated PVR of > 5 WU (area under the receiver operating characteristic curve [AUC], 0.75; *P* < .001) with an optimal cutoff of 0.48. Emphysema percentage predicted severe airflow obstruction (FEV_1_ < 50%; AUC, 0.83; *P* = .001). Using quantitative CT imaging-derived thresholds, phenotype classification demonstrated high specificity (vascular, 86.2%; parenchymal, 89.7%; mixed, 100%) with sensitivities of 46.7%, 73.3%, and 7.1%, respectively.

**Interpretation:**

Our results show that quantitative CT imaging can differentiate COPD-PH phenotypes using vascular and parenchymal markers and may serve as a noninvasive tool to characterize disease heterogeneity.

**Take-Home Points**

**Study** **Question:** Can quantitative CT scan analysis of pulmonary parenchyma and vasculature identify and distinguish various phenotypes of COPD associated with pulmonary hypertension (COPD-PH)?

**Results:** Our results show that quantitative CT imaging has the ability to use various vascular and parenchymal measures to predict pulmonary vascular resistance and FEV_1_ % predicted accurately and then use that information to predict COPD-PH phenotypes.

**Interpretation:** This study supports that quantitative CT imaging as a novel and noninvasive way to identify and phenotype COPD-PH. Dedicated research on larger cohorts is needed to establish a globally acceptable definition of these phenotypes and to validate quantitative CT imaging as a tool to diagnose various phenotypes noninvasively.

Pulmonary hypertension (PH) in patients with COPD confers worse prognosis.^1^^,^^2^ COPD associated with PH (COPD-PH) has a high prevalence overall, but severe PH is uncommon.^3^^,^^4^ The 2022 European Society of Cardiology and European Respiratory Society guidelines for the diagnosis and management of PH changed the criteria for severe PH in chronic lung disease, including COPD, to a pulmonary vascular resistance (PVR) of > 5 Wood units (WU).^5^^,^^6^ COPD-PH typically is classified as group 3 PH, but this classification may not capture fully the entire spectrum, or various phenotypes, of COPD-PH.

COPD-PH phenotypes have been defined previously using a combination of clinical, radiographic, spirometric, and pulmonary hemodynamic data obtained from right heart catheterization (RHC).^7^^,^^8^ It is of paramount importance to define various phenotypes because the phenotype carries prognostic value.^7^^,^^9^ Quantitative CT scan analysis of pulmonary vasculature and parenchyma is a novel noninvasive method to define the extent of pulmonary parenchymal and vascular disease in patients with chronic lung disease.^10^^,^^11^ It has a unique ability simultaneously to study lung parenchyma and vasculature quantitatively and has the potential ability to predict the degree of airflow obstruction (using various direct and indirect airway measurements) and PVR (using various measures of pulmonary vascular loss or remodeling).^12^, ^13^, ^14^, ^15^

Several different measurements of lung parenchymal destruction have been used in previous studies exploring quantitative CT scans. Volumetric quantitative CT imaging with appropriate software allows measurement of several airway dimensions and indices, including true orthogonal airway inner and outer diameters, wall thickness, airway areas (inner, outer, wall) and percentage of wall area (defined as the ratio of airway outer area to wall area), to the sixth or even smaller generation of airways. Additionally, parenchymal indices include percentage of low attenuation area scores at various Hounsfield units.^16^^,^^17^ Similarly, indices like total vascular volume (TVV), proportion of blood volume in pulmonary vessels with cross-sectional area of < 5 mm^2^ (BV5), proportion of blood volume in pulmonary vessels with cross-sectional area of < 10 mm^2^ (BV10), BV5 to BV10 ratio, and BV5 to TVV ratio have been used to study the pulmonary vasculature, and changes have been used to study patients with pulmonary arterial hypertension.^18^^,^^19^

We aimed to identify various phenotypes of COPD-PH, to determine their prognostic significance, and to assess whether quantitative CT imaging analysis could identify these phenotypes accurately. We hypothesized that transplant-free survival differs among COPD-PH phenotypes and that quantitative CT imaging analysis can identify phenotypes and prognosticate outcomes in COPD-PH effectively.

## Study Design and Methods

### Study Design and Patient Selection

This is a single-center observational study examined retrospective cohort of patients with COPD who underwent clinically indicated RHC for suspected or confirmed PH at a large transplant referral center (Temple University Hospital) from January 2011 through December 2023. Patients with COPD and precapillary PH were included. COPD was defined and airflow limitation was categorized according to the Global Initiative for Chronic Obstructive Pulmonary Disease parameters, whereas precapillary PH was defined as mean pulmonary artery pressure (mPAP) of > 20 mm Hg, pulmonary capillary wedge pressure of ≤ 15 mm Hg, and PVR of > 2 WU on RHC. Patients with combined pulmonary fibrosis and emphysema, diagnosed based on clinical documentation and radiographic and pulmonary function test data, were excluded. Patients with postcapillary PH (pulmonary capillary wedge pressure > 15 mmHg) or multifactorial PH also were excluded. Severity of airflow obstruction, as measured by FEV_1_ % predicted after bronchodilator administration, was used to divide patients into 2 categories: those with FEV_1_ of < 50% predicted and those with FEV_1_ of > 50% predicted.

For hemodynamic classification, all patients were classified into 2 categories: PVR of < 5 WU and PVR of > 5 WU. Airflow obstruction and PVR severity were used to generate 3 COPD-PH phenotypes (Table 1): pulmonary vascular phenotype (FEV_1_ > 50% predicted and PVR > 5 WU), pulmonary parenchymal phenotype (FEV_1_ < 50% predicted and PVR < 5 WU), and mixed pulmonary vascular and parenchymal phenotype (FEV_1_ < 50% predicted and PVR > 5 WU). All patients underwent baseline full pulmonary function testing, the 6-minute walk test, echocardiography, CT imaging of the thorax, and RHC.

**Table 1:** Classification of Patients With COPD-PH Into Different Phenotypes Based on FEV_1_ % Predicted and Pulmonary Vascular Resistance

Variable	Pulmonary Vascular Phenotype	Mixed Phenotype	Pulmonary Parenchymal Phenotype
PVR, WU	≥ 5	≥ 5	< 5
FEV_1_, % predicted	> 50	< 50	< 50

COPD-PH = COPD associated with pulmonary hypertension; PVR = pulmonary vascular resistance; WU = Wood unit.

Volumetric and pulmonary vascular reconstruction analyses were performed using Lung Print software (VIDA Diagnostics). Thirty patients were evenly but randomly selected from each of the 3 phenotype groups and de-identified. Axial CT scans of the thorax closest to the initial diagnostic RHC were analyzed to calculate TVV and vessel volume for areas of < 5 mm^2^ and 10 mm^2^ (to determine BV5 and BV10), regional lung volume, total emphysema volume, and emphysema percentage. Our study met with approval for a waiver of informed consent according to the Western Institutional Review Board Research Integrity and Compliance (Identifier: 31795). Procedures were followed in accordance with the ethical standards of the Western Institutional Review Board and the tenets of the Helsinki Declaration of 1975.

### Statistical Analysis

Statistical analysis was performed by comparing 3 groups of phenotypes. All continuous variables were presented as the mean (SD) unless stated otherwise. Categorical variables were compared using the Pearson χ^2^ test or Fisher exact test where applicable. Continuous variables were compared between groups using the paired *t* test. First, Kaplan-Meier survival analysis was performed to compare survival probability among the clinical phenotypes. Cox regression was performed to determine the risk of death before potential lung transplantation among the phenotypes. Second, the mean ratios between various vascular (BV5, BV10, TVV, and BV5 ratio) and parenchymal (mean percentage emphysematous lung parenchyma) indices obtained from quantitative CT imaging analysis were calculated and compared among phenotypes using the analysis of variance. Third, receiver operator characteristic curve analysis was performed to assess the ability of BV5 to TVV ratio to predict severe PH (PVR > 5 WU) and the ability of emphysema percentage to predict FEV_1_ of < 50% predicted. Fourth, using quantitative CT imaging cutoffs, the sensitivity and specificity of quantitative CT imaging to differentiate between various COPD-PH phenotypes were calculated. Finally, Kaplan-Meier survival analysis was performed to compare the survival probabilities among the quantitative CT imaging phenotypes, and comparison was made to clinical phenotypes. The statistical analysis was performed using IBM SPSS Statistics version 29 and GraphPad Prism software (formerly GraphPad Software, Inc.).

## Results

A total of 480 patients with coexisting COPD and PH were screened to be included in the study (Fig 1). Of these, 53 patients had combined pulmonary fibrosis and emphysema and 111 patients showed evidence of World Health Organization group II PH with elevated pulmonary capillary wedge pressure (> 15 mm Hg); these patients were excluded from the study. A total of 316 patients fulfilled the inclusion and exclusion criteria. Of these 316 patients, 30 patients demonstrated the vascular phenotype, 61 patients demonstrated the mixed phenotype, and 225 patients demonstrated the parenchymal phenotype. Of these patients, 30 patients from each phenotype group were selected equally and randomly to be included in the final analysis.

**Figure 1:**
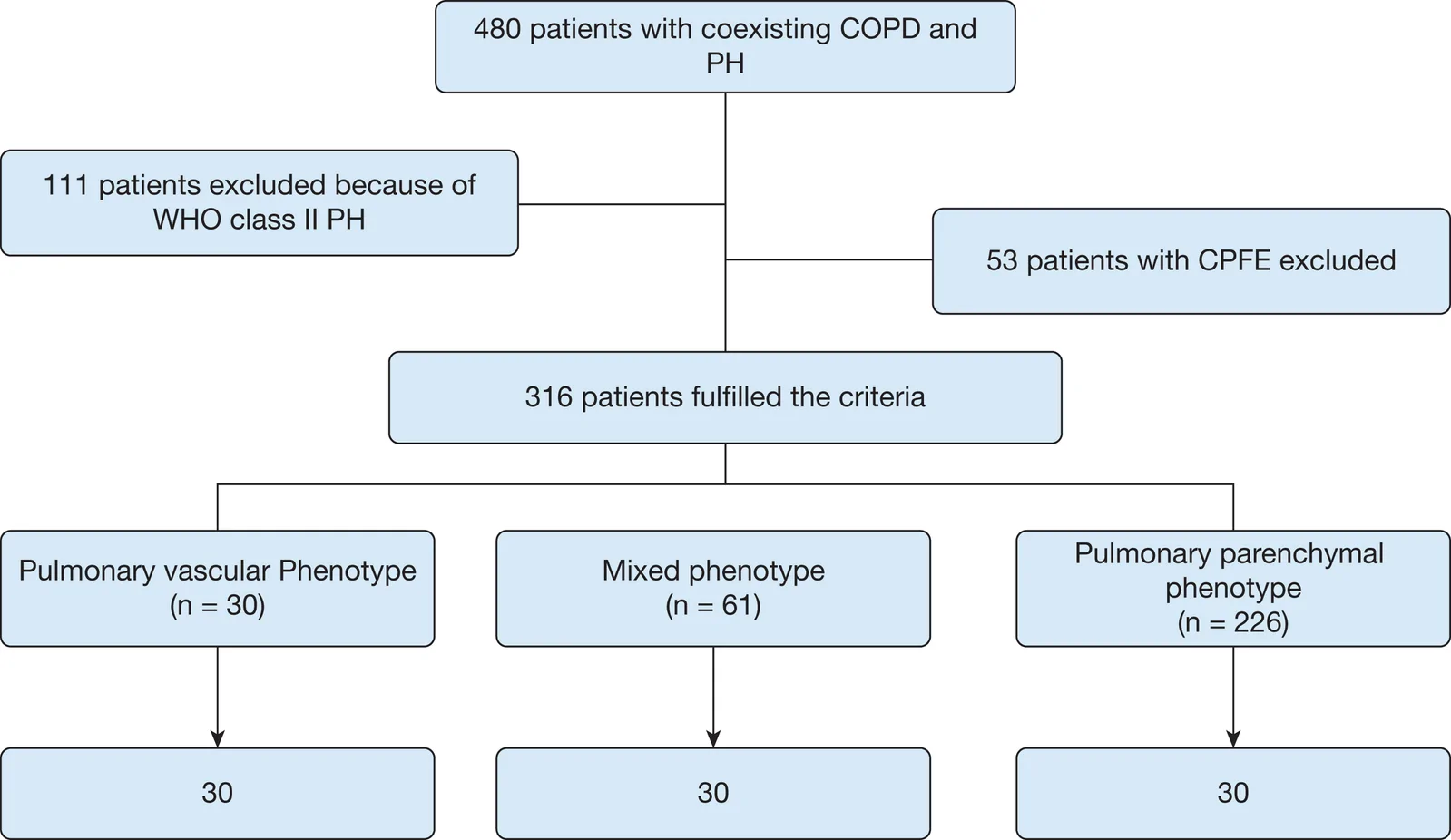
Patient cohort flowsheet. Patients with multifactorial PH, WHO group II PH with evidence of elevated pulmonary capillary wedge pressure, or both were excluded. Additionally, patients with radiographic and spirometric documented evidence of combined pulmonary fibrosis and emphysema CPFE also were excluded. In the overall cohort, most patients demonstrated the pulmonary parenchymal phenotype, defined as pulmonary vascular resistance of 2 to 5 Wood units and FEV_1_ of < 50% predicted. CPFE = combined pulmonary fibrosis and emphysema; PH = pulmonary hypertension; WHO = World Health Organization.

Baseline characteristics of patients within each phenotype closest to the diagnostic RHC are shown in Table 2. The mean ages of the 3 groups were comparable, with a slightly younger patient cohort in the pulmonary parenchymal group. All phenotypes predominantly consisted of the female patient population. The pulmonary vascular phenotype (pulmonary arterial hypertension phenotype) showed the most profound hemodynamic abnormalities, with significantly more elevated mPAP and PVR, compared with the pulmonary parenchymal phenotype.

**Table 2:** Baseline Characteristics of Various COPD-PH Phenotypes

Variable	PAH Phenotype (n =30)	Mixed Phenotype (n =30)	Parenchymal Phenotype (n =30)
Age, y	67.4 (8.8)	65.2 (8.06)	63.6 (7.1)
Sex, male/female	14/16	13/17	14/16
Systolic mPAP, mm Hg^a^	71.5 (15.6)	60.8 (16.5)	38.2 (8.0)
mPAP, mm Hg^a^	44.3 (9.7)	39.5 (10.3)	25.9 (5.2)
Mean PCWP, mm Hg^a^	12.4 (3.1)	10.8 (3.7)	13.2 (3.1)
Mean PVR^a^	9.5 (3.5)	7.5 (2.1)	2.8 (0.54)
Mean FEV_1_, % predicted^a^	68.5 (12.3)	35.4 (4.6)	22.3 (5.5)
Mean Dlco, % predicted	26 (5.2)	24.2 (3.9)	23.2 (2.8)
Mean oxygen requirement, L^a^	7.6 (5.1)	4.7 (3.7)	4.2 (2.9)
Mean 6MWD, m	228 (101.6)	208.7 (91.6)	215.5 (84.6)

Data are presented as No. or mean (SD). 6MWD = 6-minute walk distance; COPD-PH = COPD associated with pulmonary hypertension; Dlco = diffusion capacity of the lungs for carbon dioxide; mPAP = mean pulmonary artery pressure; PCWP = pulmonary capillary wedge pressure; PVR = pulmonary vascular resistance.

a Significant difference found (*P* < .05).

The mean PVR in the pulmonary vascular phenotype was 9.5 WU, compared with 7.5 WU for the mixed phenotype and 2.8 WU for the pulmonary parenchymal phenotype. The mean FEV_1_ % predicted for the above phenotypes was 67.4%, 35.8%, and 22.3%, respectively, showing very severe airflow obstruction in the pulmonary parenchymal phenotype. Mean diffusion capacity of the lungs for carbon dioxide was severely reduced in all phenotype groups with no significant intergroup difference. Patients with the PAH phenotype showed greater hypoxia and, on average, required more oxygen. Figure 2 depicts the relationships among various COPD-PH phenotypes.

**Figure 2:**
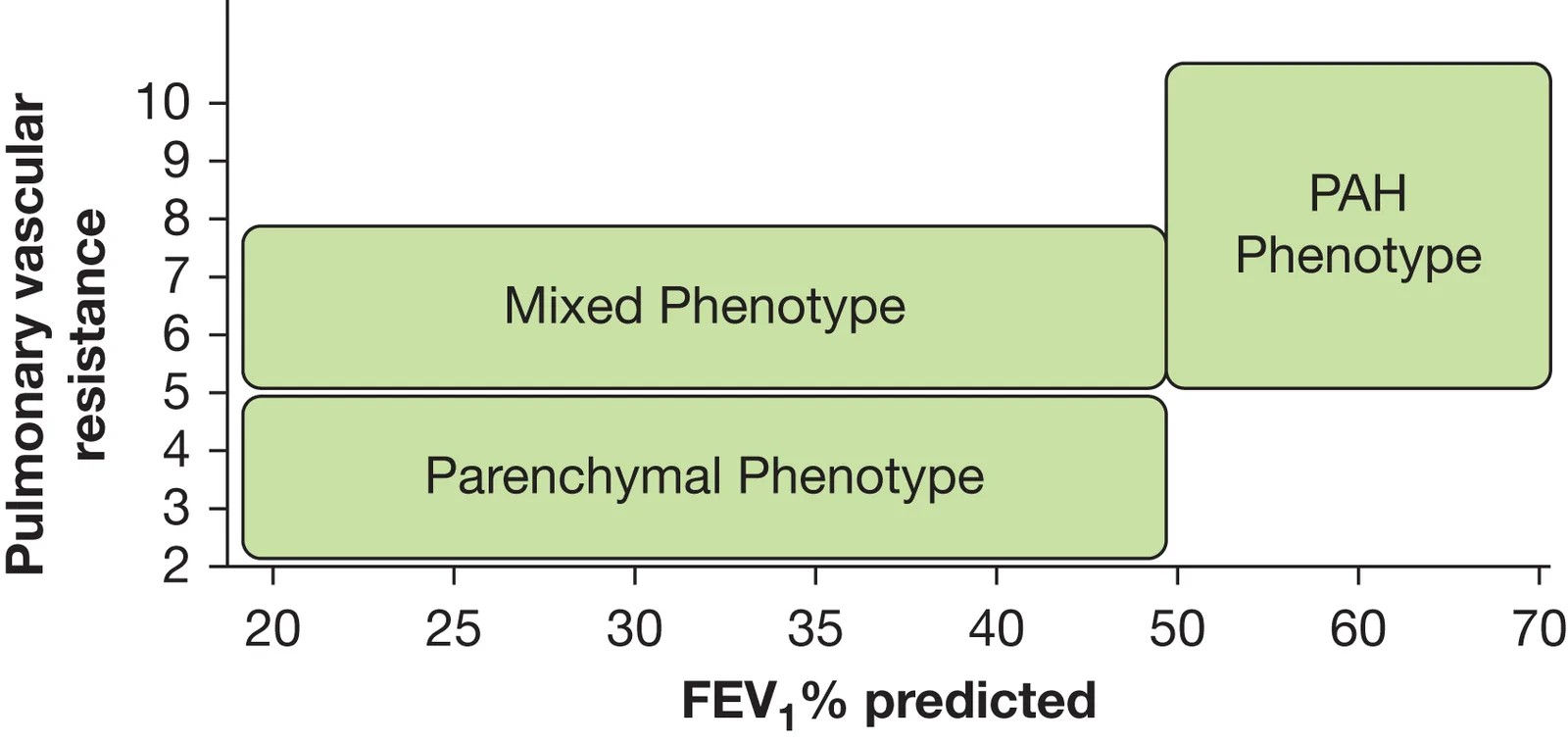
Graphical representation of various phenotypes of COPD associated with pulmonary hypertension with their respective relationship with pulmonary vascular resistance (in Wood units) and FEV_1_ % predicted. PAH = pulmonary arterial hypertension.

In clinical phenotype analysis, 14.2% of patients (n = 4) died in the pulmonary vascular group before a potential lung transplant. In the mixed phenotype group, 31% of patients (n = 9) died before a potential lung transplant. In the pulmonary parenchymal group, 6.67% of patients (n = 2) died before a potential lung transplant. Kaplan-Meier survival analysis showed significantly different survival probabilities among the 3 phenotypes, with the mixed pulmonary vascular and parenchymal phenotypes demonstrating the worst survival probability (*P* = .04, log-rank test) (Fig 3).

**Figure 3:**
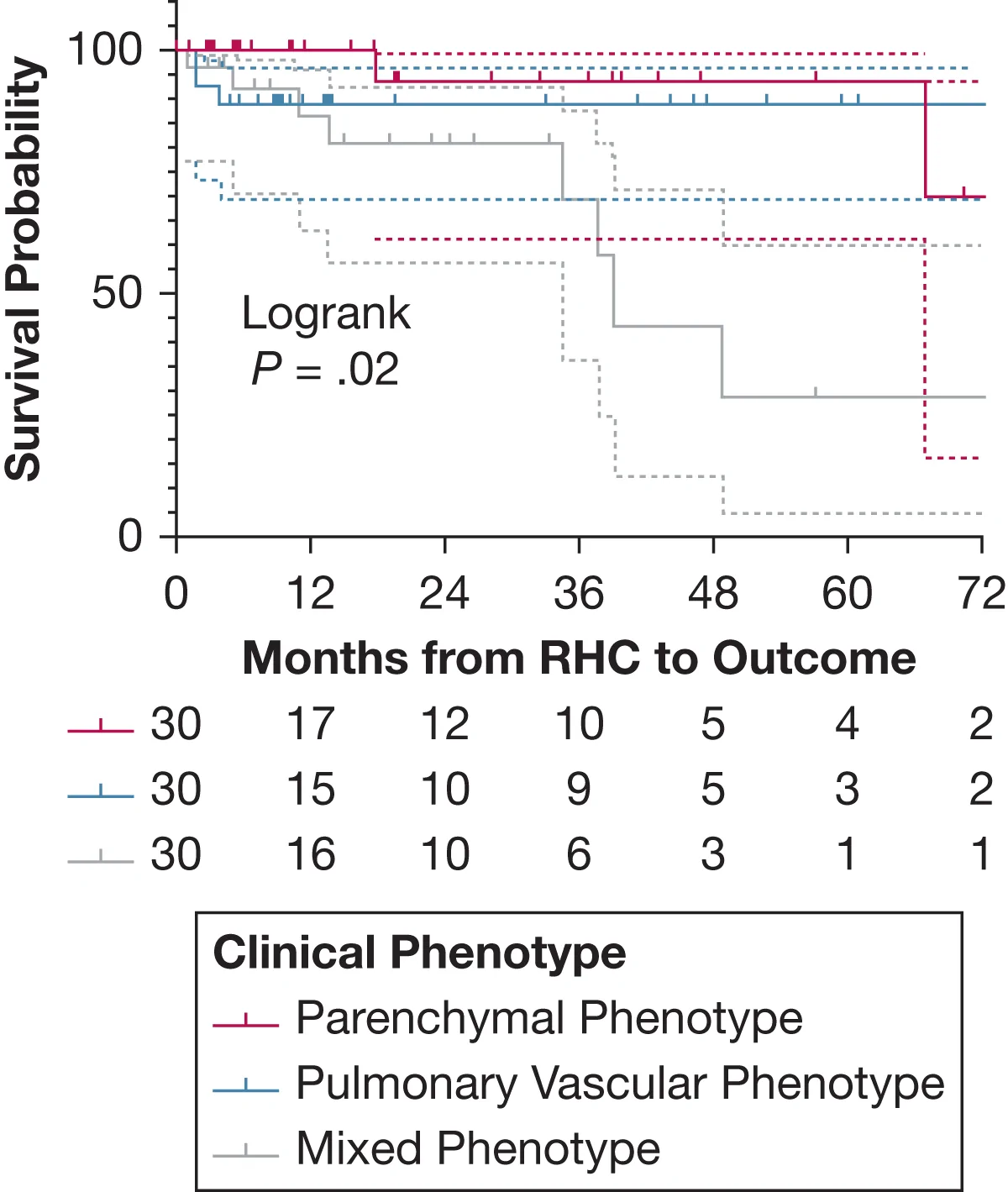
Kaplan-Meier survival curve showing survival probabilities among the 3 clinical phenotypes of COPD associated with pulmonary hypertension. The numbers of patients at risk are displayed at the bottom. Changes in the graph resulting from death before transplantation while also censoring those who underwent lung transplantation because they would no longer be at risk of death before transplantation. Lung transplantation is not reflected in the actual Kaplan-Meier curve. A significant difference is seen, with lower survival probabilities in the mixed phenotype compared with the parenchymal phenotype, before potential lung transplantation (hazard ratio, 5.74; 95% CI, 1.21-27.32; *P* = .03). The dotted lines represent the CIs for the survival curves. RHC = right heart catheterization.

Cox regression showed that compared with the parenchymal phenotype, the mixed phenotype was associated significantly with an increased risk of mortality before potential lung transplantation (OR, 5.74; 95% CI, 1.21-27.32; *P* = .03). Compared with the parenchymal phenotype, the pulmonary vascular phenotype showed an increased risk of mortality (OR, 1.61; 95% CI, 0.27-9.72; *P* = .61), but this was not statistically significant.

Given that the survival curves in Figure 3 crossed, we tested the Cox regression model for the assumption of proportional hazards via a log-minus-log plot. The log-minus-log plot for the clinical phenotypes shows parallel lines for the 3 phenotypes (e-Fig 1), suggesting that the assumption of proportional hazards is not violated and that the risk of death associated with the clinical phenotypes remains constant over time.

Next, vascular reconstruction analysis was performed using quantitative CT imaging, which showed that the mean BV5 was 62.9 mm^2^ for the vascular phenotype, 99.9 mm^2^ for the parenchymal phenotype, and 72.8 mm^2^ for the mixed phenotype (*P* < .001). The mean TVV was 130.0 cm^3^ for the vascular phenotype, compared with 169.7 cm^3^ for the parenchymal phenotype and 139.8 cm^3^ for the mixed phenotype (*P* < .001). Figure 4 shows a single-slice (coronal view) CT scan with vascular reconstruction images showing vascular and parenchymal features while comparing 1 patient from each category of the 3 phenotypes.

**Figure 4:**
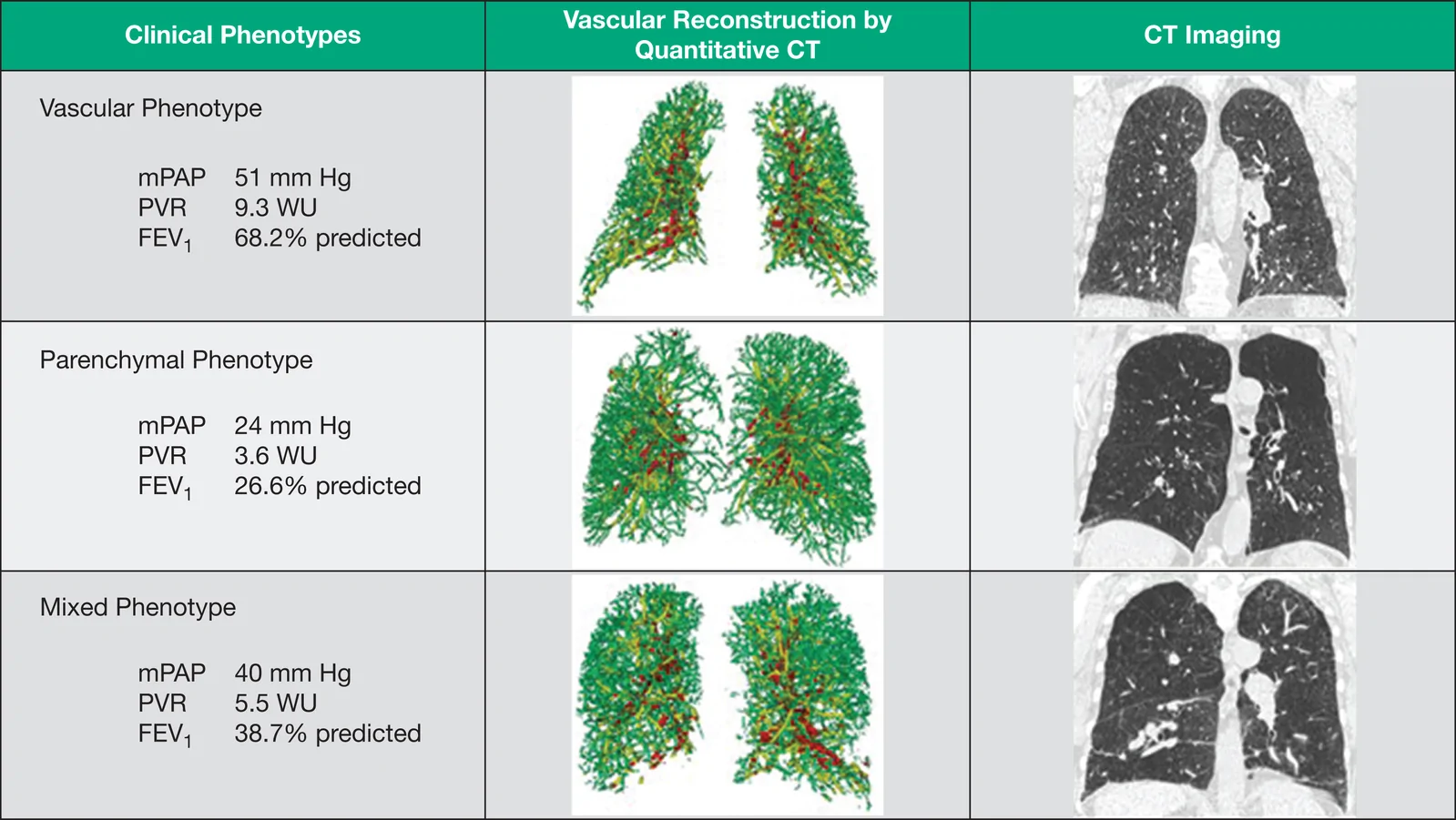
Clinical characteristics, CT imaging, and vascular reconstruction from 1 patient from each phenotype group. The vascular phenotype showed the most loss of the proportion of blood volume in pulmonary vessels with cross-sectional area of < 5 mm^2^ (green) compared with both the parenchymal and mixed phenotypes. On coronal slice CT imaging, more destructive emphysema can be appreciated in the patient with the parenchymal phenotype. mPAP = mean pulmonary artery pressure; PVR = pulmonary vascular resistance; WU = Wood unit.

The pulmonary parenchymal phenotype showed the highest mean percentage of emphysematous lung parenchyma (46.8%), followed by 29.2% for the mixed phenotype and 17.2% for the vascular phenotype (*P* < .001). Table 3 summarizes the quantitative CT imaging parameter differences across phenotypes.

**Table 3:** Quantitative CT Scan Indices of Pulmonary Vasculature and Parenchyma

Variable	PAH Phenotype (n = 30)	Mixed Phenotype (n =30)	Parenchymal Phenotype (n =30)	*P* Value
Total vessel volume, mL	130.0 (28.4)	139.8 (21.9)	169.7 (36.8)	< .001
BV5, mm^3^	62.9 (31.3)	72.8 (32.6)	99.9 (18.5)	< .001
BV10, mm^3^	23.5 (12.5)	26.1 (16.2)	45.3 (16)	< .001
BV5/BV10 ratio	0.48 (0.07)	0.52 (0.10)	0.58 (0.98)	< .001
Emphysema volume, cm^3^	611 (349)	1,304 (544)	2,458 (1,077)	< .001
% LAA				
At –950 HU	13.2 (10.8)	24.2 (14.7)	36.8 (10.6)	< .001
At –910 HU	21.8 (14.6)	37.7 (19.3)	55.4 (10.9)	< .001

Data are presented as mean (SD) unless otherwise indicated. BV5 = proportion of blood volume in pulmonary vessels with cross-sectional area of < 5 mm^2^; BV10 = proportion of blood volume in pulmonary vessels with cross-sectional area of < 10 mm^2^; HU = Hounsfieldy unit; LAA = low attenuation area.

The BV5 to TVV ratio calculated via quantitative CT imaging analysis significantly predicted PVR of < 5 WU in the receiver operator characteristic curve analysis (*P* < .001; area under the receiver operating characteristic curve, 0.75), with an optimal ratio cutoff of 0.484. A BV5 ratio of < 0.484 were associated significantly with increased risk of PVR of > 5 WU developing compared with the higher ratio (OR, 3.78; 95% CI, 1.41-10.18; *P* = .01) (Fig 5).

**Figure 5:**
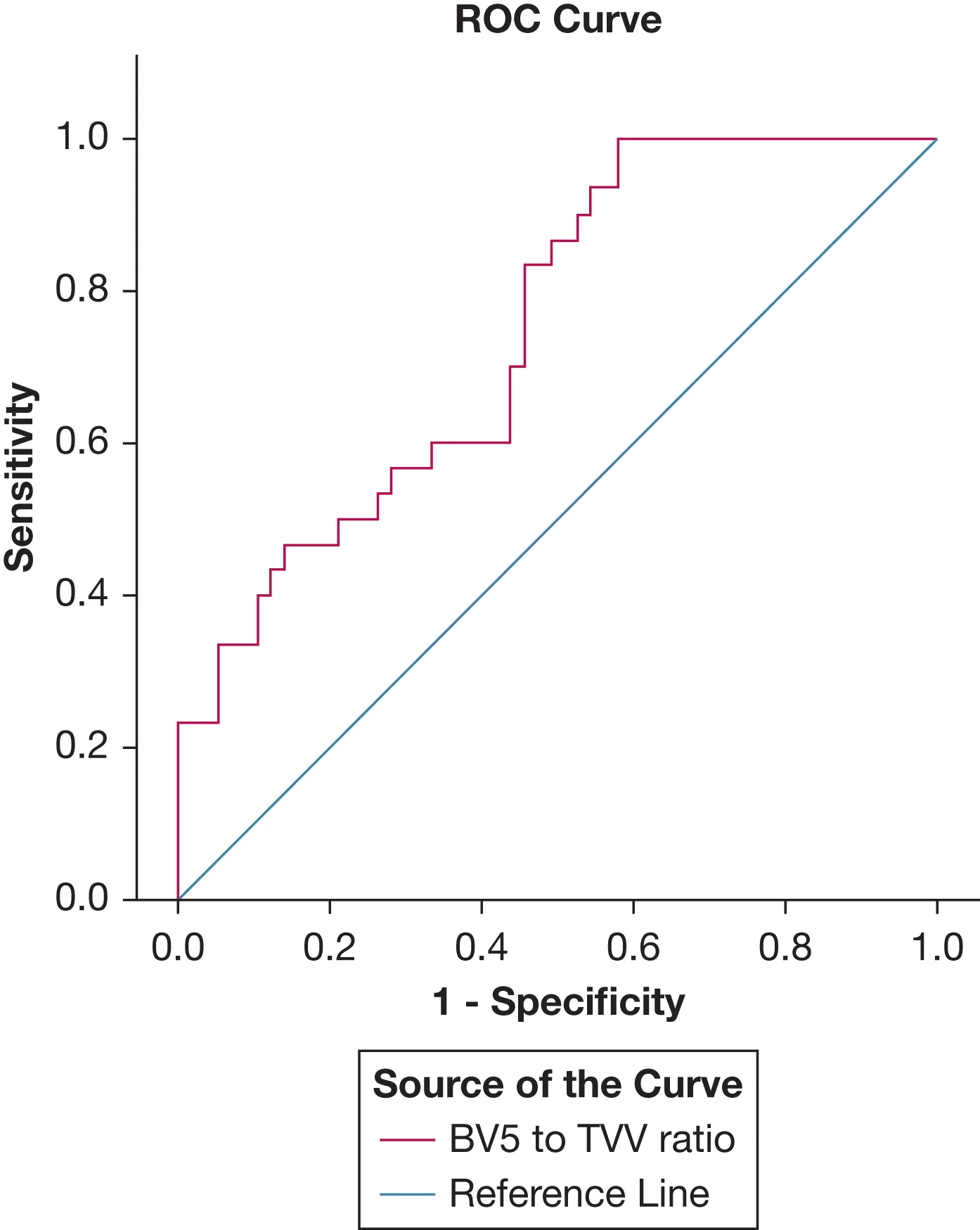
ROC curve showing BV5 to TVV ratio predicting pulmonary vascular resistance. BV5 = proportion of blood volume in pulmonary vessels with cross-sectional area of < 5 mm^2^; ROC = receiver operating characteristic; TVV = total vascular volume.

The Brier calibration score for the BV5 ratio predicting PVR of > 5 WU is 0.0. The Brier calibration score for the percentage of emphysema predicting FEV_1_ of < 50% is 0.1. These findings suggest good calibration and accuracy of the BV5 ratio predicting PVR of > 5 WU and good calibration and accuracy of emphysema percentage predicting FEV_1_ of < 50% predicted.

In the parenchymal analysis, emphysema percentage effectively predicted severe airflow obstruction (FEV_1_ < 50%; *P* = .001; area under the receiver operating characteristic curve, 0.83). The optimal emphysema percentage cutoff for this degree of airflow obstruction, as measured by FEV_1_ % predicted, was 22.28%. An emphysema percentage of > 22.28% was associated significantly with the risk of having an FEV_1_ of < 50% in logistic regression (OR, 14.82; 95% CI, 4.47-49.17; *P* < .001) (Fig 6).

**Figure 6:**
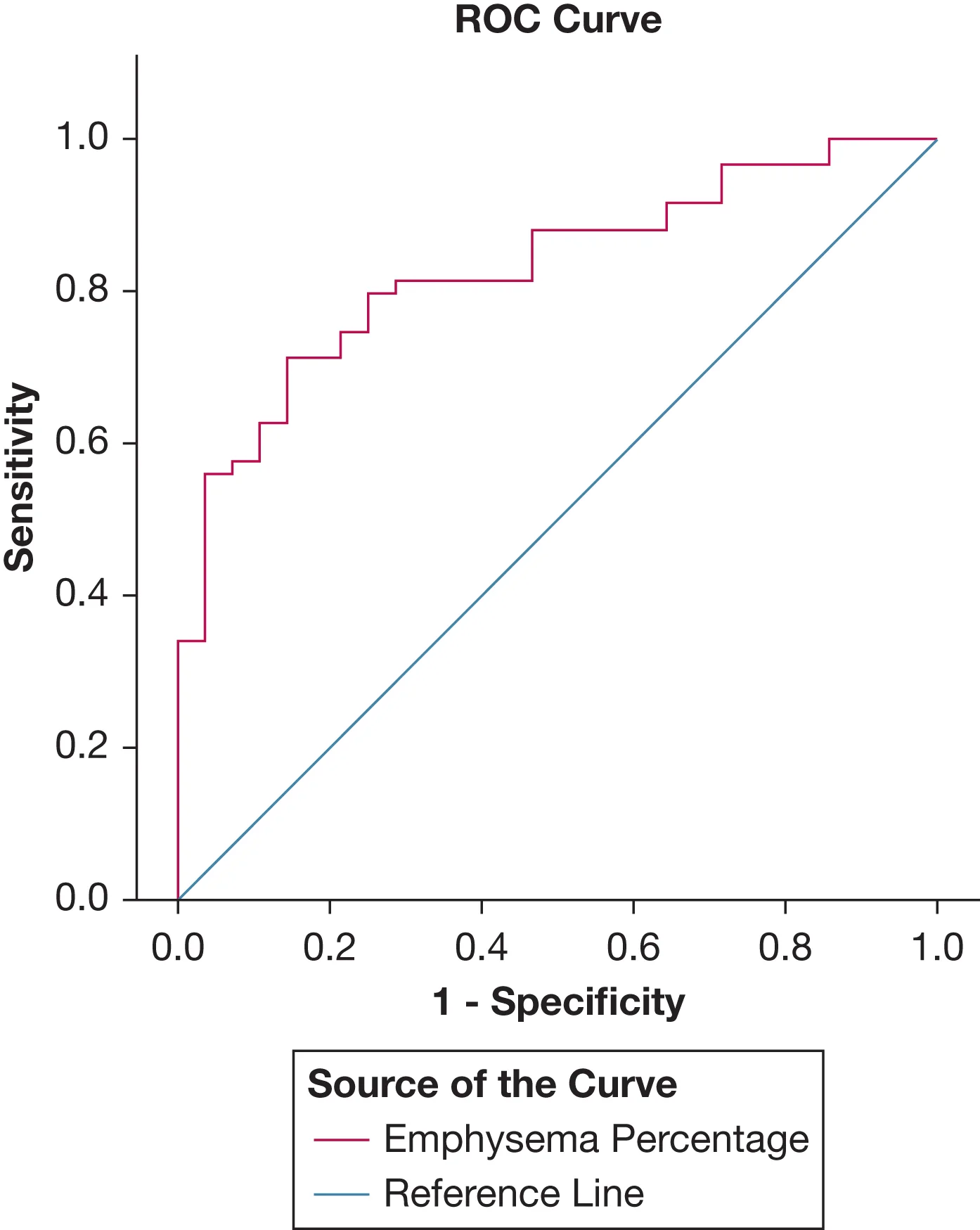
ROC curve showing emphysema percentage predicting FEV_1_ % of < 50%. ROC = receiver operating characteristic.

Using the quantitative CT cutoffs, the analysis demonstrated high specificity (86.4%, 98.3%, and 100%) for identifying the 3 phenotypes: pulmonary vascular, parenchymal, and mixed, with corresponding sensitivities of 42.9%, 90%, and 10.3%, respectively. By quantitative CT imaging analysis, a total of 42 patients were identified as demonstrating the parenchymal phenotype, 20 patients were identified as demonstrating the pulmonary vascular phenotype, and 4 patients were identified as demonstrating the mixed phenotype. Analysis showed similar results to clinical phenotype data, with the mixed phenotype having the worst mortality (75%; n = 3) compared with the pulmonary vascular and parenchymal phenotypes (n = 5 [25%] and n = 3 [7.1%], respectively) and worse survival probability in Kaplan-Meier analysis (Fig 7). Cox regression showed that compared with the quantitative CT imaging-identified parenchymal phenotype, the quantitative CT imaging-identified mixed phenotype was associated significantly with increased risk of death before transplantation (OR, 11.37; 95% CI, 2.16-59.90; *P* = .004), whereas also an increased risk of death before transplant was associated with the pulmonary vascular phenotype (OR, 3.03; 95% CI, 0.67-13.71; *P* = .15), although this was not statistically significant.

**Figure 7:**
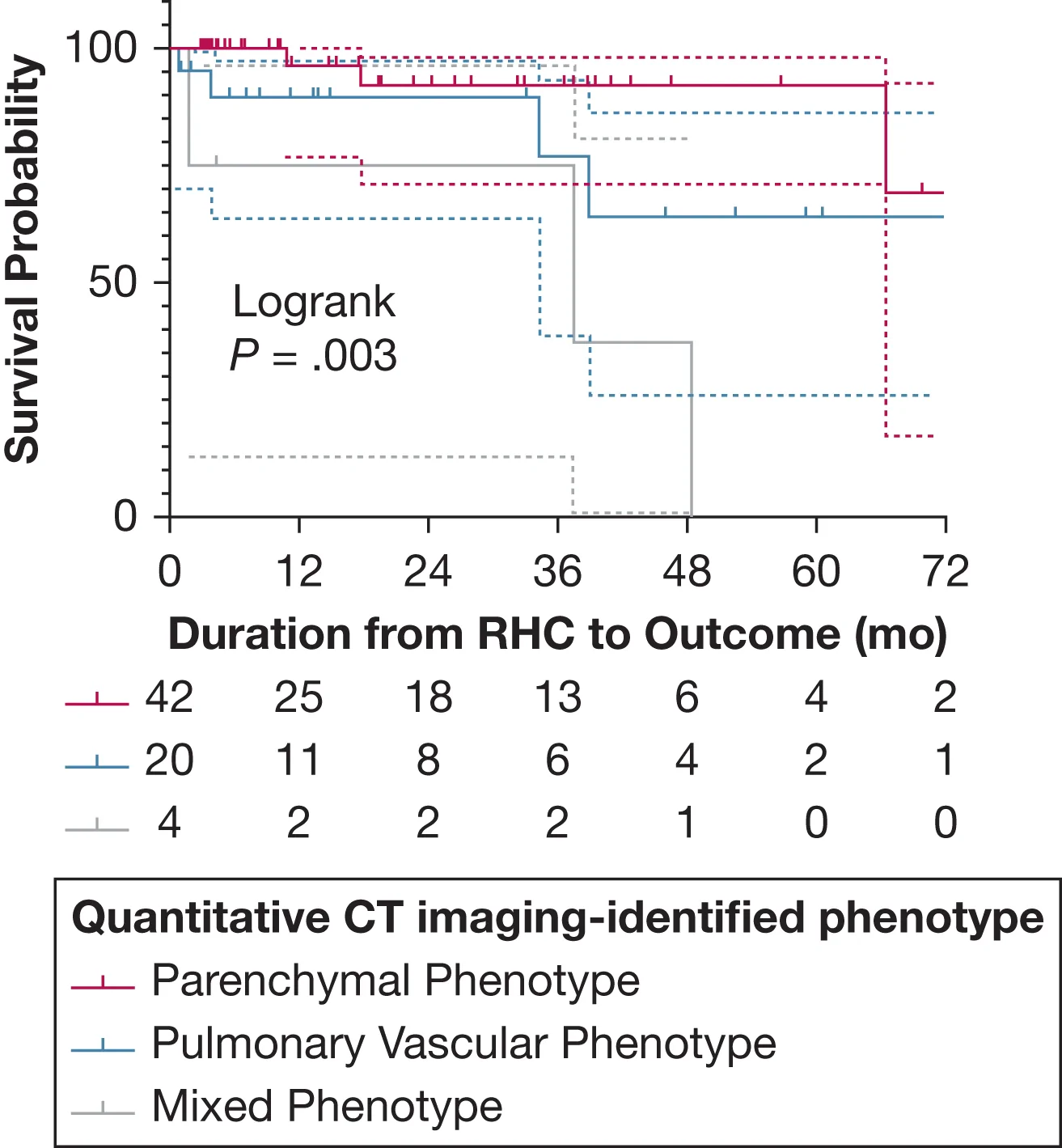
Kaplan-Meier survival curve showing survival probabilities among the 3 quantitative CT imaging phenotypes. Again, the numbers of patients at risk are displayed at the bottom. Changes in the graph resulting from death before transplantation while also censoring those who underwent lung transplantation, because they would no longer be at risk of death, before transplantation. Lung transplantation is not reflected in the actual Kaplan-Meier curve. Cox regression showed that compared with the quantitative CT imaging-identified parenchymal phenotype, the quantitative CT imaging-identified mixed phenotype was associated significantly with increased risk of death before transplantation (hazard ratio, 11.37; 95% CI, 2.16-59.90; *P* = .004). The dotted lines represent the CIs for the survival curves. RHC = right heart catheterization.

## Discussion

In our study, quantitative CT scan analysis was able to predict, differentiate, and prognosticate various COPD-PH phenotypes in patients with COPD and PH. To determine these phenotypes using quantitative CT imaging alone, we first used the BV5 ratio (BV5 to TVV) to predict the PVR, and based on our study, a cutoff of 0.48 was used to differentiate between PVR of > 5 WU and PVR of < 5 WU. This binary distinction of PVR was based on the latest European Society of Cardiology and European Respiratory Society guidelines, because it differentiates between severe and nonsevere PH. This then was combined with emphysema percentage analysis (to estimate FEV_1_ % predicted) to generate quantitative CT imaging phenotypes. Using these cutoffs, quantitative CT imaging analysis showed high specificity for predicting these phenotypes. To the best of our knowledge, this is the first predictive model to predict COPD-PH phenotypes accurately using quantitative CT imaging data alone.

Next, quantitative CT imaging-generated phenotypes were analyzed and showed similar survival data to clinical phenotypes. As seen in clinical phenotypes, it showed worse outcomes in the mixed phenotype compared with the parenchymal or vascular phenotypes. The ability of quantitative CT imaging to predict and prognosticate these phenotypes accurately using noninvasive measures alone is a major and important scientific discovery that can be an extremely valuable tool in identifying such patients and implementing personalized management plans. Although quantitative CT imaging has been used to study vascular and parenchymal abnormalities in patients with COPD and patients with pulmonary arterial hypertension,^15^^,^^20^^,^^21^ its use to predict survival outcomes in patients with COPD and PH is a novel concept and has not been reported before. Survival data from clinical phenotypes are consistent with the work of Kovacs et al, which showed the worst survival in patients with coexistant severe PH and severe COPD.^7^^,^^22^ Our group previously demonstrated quite similar results using a large cohort of patients with COPD-PH and separating them based on the already mentioned phenotype criteria. Three hundred seventeen patients were separated into vascular (n = 30), parenchymal (n = 226), and mixed (n = 61) phenotypes. Kaplan-Meier survival analysis showed significantly different survival probabilities among the 3 phenotypes, with the mixed phenotype demonstrating lower survival probability (*P* = .04, log-rank test). Cox regression showed that compared with the parenchymal and vascular phenotypes, the mixed phenotype was associated significantly with an increased risk of mortality (OR, 2.12; 95% CI, 1.17-3.86; *P* = .001).^23^

Distinct phenotypes of PH in COPD (COPD-PH) have been mentioned and discussed in studies previously.^24^, ^25^, ^26^, ^27^, ^28^ Similarly, various cutoffs to gauge the degree of airflow obstruction and severity of PH also have been described.^7^^,^^8^^,^^29^ These cutoffs are based on expert opinion, primarily based on the underlying pathophysiologic mechanisms of PH in patients with COPD. So far, to the best of our knowledge, no universally acceptable cutoffs or guidelines are available to define various COPD-PH phenotypes. We chose to separate patients with COPD-PH into 3 distinct phenotypes to help study them in detail and to elicit comparisons. This decision was intentional and with an acknowledgment that PH in COPD is more of a spectrum, rather than buckets of a few groups.^26^^,^^30^, ^31^, ^32^ We used a PVR of > 5 WU to determine severe PH and the pulmonary vascular phenotype; this group showed severely elevated mPAP, severely elevated PVR, mild to moderate airflow obstruction, and the most hypoxemia, despite the least emphysema. This group is quite important, because this phenotype is hypothesized to be most likely to respond to pulmonary vasodilator treatment. When studied with quantitative CT imaging, this phenotype demonstrated low mean BV5 compared with the parenchymal phenotype. This is quite an important finding that points to greater small pulmonary vessel destruction in severe PH compared with PH related to destructive emphysema. BV5 in the mixed phenotype is quite close to that in the vascular phenotype, likely because of the comparable severity of PH, and BV5 was highest in the parenchymal phenotype. In contrast, the pulmonary parenchymal phenotype represents patients with severe or end-stage COPD with the diagnosis of PH according to the European Society of Cardiology and European Respiratory Society criteria (PVR > 2 WU). These patients showed severe to very severe airflow obstruction, the most amount of emphysema percentage, the lowest PVR on RHC, and the least elevated mPAP. These patients most likely share the same mechanism of PH owing to severe destructive emphysema, resulting in the loss of alveolar capillary structure, and likely will demonstrate worsening hypoxemia with pulmonary vasodilator treatment as a result of ventilation-perfusion mismatch. On quantitative CT imaging analysis, they showed relatively preserved BV5 and the highest percentage of emphysema. The mixed phenotype included a mix of characteristics from both the pulmonary vascular and parenchymal groups. This group is the most complicated and the least studied and can demonstrate variable responses to pulmonary vasodilators.

In our study, abnormalities in BV5 were similar to what was reported by Cajigas et al,^19^ but differed in terms of the subgroup analysis of patients with COPD-PH, in whom they did not find any difference in patients with mild to moderate PH and patients with severe PH. This is likely related to the small individual subgroup sample size in their study. Additionally, patients with the parenchymal phenotype showed a higher mean TVV compared with the other 2 phenotype groups. This is also consistent with the findings of Elbehairy et al, who demonstrated higher TVV in patients with nonsevere PH.^20^^,^^21^^,^^33^, ^34^, ^35^ Quantitative CT imaging vascular and parenchymal measures were obtained from the inspiratory CT scans, but without the confirmation of full inspiration at total lung capacity. Theoretically, this could have some impact on vascular measurements, and it was an inherent limitation of the retrospective study. In rare cases when both inspiratory and expiratory CT scans were available, we preferred inspiratory images over expiratory ones for quantitative CT imaging analysis. We then used the BV5 to TVV ratio to predict PVR of > 5 WU. This can be quite significant in the clinical determination of patients with greater pulmonary vascular involvement, resulting in severe PH. Similarly, in quantitative CT imaging analysis of lung parenchyma, the emphysema index was used in 1 study in which it seemed to correlate well with the degree of airflow obstruction (FEV_1_ % predicted), but has not been used to predict FEV_1_ and create phenotypes.

In the present cohort, quantitative CT scan analysis showed high specificity to detect mixed pulmonary and parenchymal phenotypes using vascular and parenchymal measures, but with very low sensitivity. This is likely related to advanced parenchymal and vascular abnormalities in such patients, so that quantitative CT imaging cannot rule out the probability of the presence of the mixed phenotype. Inherent to the nature of this phenotype, significant overlap with vascular and parenchymal features of the other two phenotypes exists, which makes it harder not only to characterize this ,phenotype but also to exclude its presence.

### Study Limitations

Our study has some important limitations. First, this was a single-center observational study carried out on a retrospective cohort, which limits generalizability. Second, the novel combination of FEV_1_ % predicted and PVR used to generate 3 phenotypes currently is arbitrary and has not been validated in larger studies. Future prospective multicenter studies using quantitative CT imaging and targeting specific phenotypes are necessary to universalize and clarify the significance of inferred measures in this study. Third, despite our best efforts in trying and using the chest CT scans devoid of any large consolidations, effusions, masses, or confounding opacities that could affect quantitative CT imaging analysis, we could not make sure that the analyzed scans were obtained at full inspiration or total lung capacity. Theoretically, this could have affected vessel volume calculation in some patients.

## Interpretation

Our results show that patients with COPD-PH demonstrate distinct phenotypes that differ from each other in terms of clinical characteristics, survival, and potential response to treatment. Patients with severe pulmonary vascular disease and moderate to severe airway or pulmonary parenchymal disease had the worst prognosis, likely related to coexisting severe disease, poor understanding of this phenotype, and lack of treatment guidelines. Various quantitative CT imaging indices of pulmonary vascular and parenchymal analysis can be extremely valuable in noninvasively characterizing these phenotypes using prediction models, which can help to prognosticate and alter the management plans for these patients.

## Funding/Support

The authors have reported to *CHEST Pulmonary* that no funding was received for this study.

## Financial/Nonfinancial Disclosures

None declared.

## Figures and Tables

**Figure S1:**
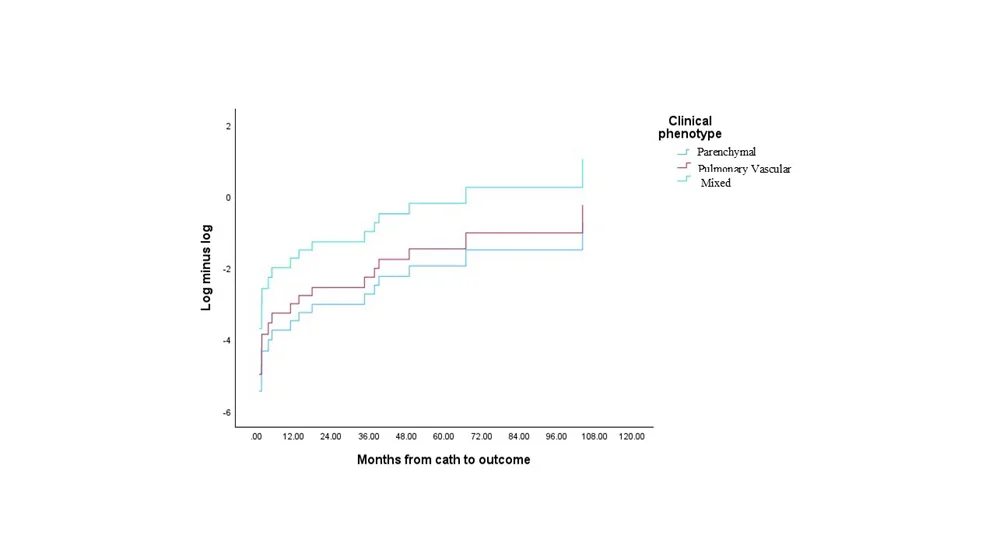


## References

[1] Gall H., Felix J.F., Schneck F.K. (2017). The Giessen Pulmonary Hypertension Registry: survival in pulmonary hypertension subgroups. J Heart Lung Transplant.

[2] Zeder K., Avian A., Bachmaier G. (2021). Elevated pulmonary vascular resistance predicts mortality in COPD patients. Eur Respir J.

[3] Andersen K.H., Iversen M., Kjaergaard J. (2012). Prevalence, predictors, and survival in pulmonary hypertension related to end-stage chronic obstructive pulmonary disease. J Heart Lung Transplant.

[4] Zhang L., Liu Y., Zhao S. (2022). The incidence and prevalence of pulmonary hypertension in the COPD population: a systematic review and meta-analysis. Int J Chronic Obstruct Pulmon Dis.

[5] Nathan S.D., Barbera J.A., Gaine S.P. (2019). Pulmonary hypertension in chronic lung disease and hypoxia. Eur Respir J.

[6] Humbert M., Kovacs G., Hoeper M.M. (2023). 2022 ESC/ERS guidelines for the diagnosis and treatment of pulmonary hypertension. Eur Respir J.

[7] Kovacs G., Avian A., Bachmaier G. (2022). Severe pulmonary hypertension in COPD: impact on survival and diagnostic approach. Chest.

[8] García A.R., Piccari L. (2022). Emerging phenotypes of pulmonary hypertension associated with COPD: a field guide. Curr Opin Pulmon Med.

[9] Steger M., Canuet M., Enache I. (2024). Survival and response to pulmonary vasodilator therapies in patients with chronic obstructive pulmonary disease and pulmonary vascular phenotype. Respir Med.

[10] Chen A., Karwoski R.A., Gierada D.S., Bartholmai B.J., Koo C.W. (2020). Quantitative CT analysis of diffuse lung disease. Radiographics.

[11] Silva M., Milanese G., Seletti V., Ariani A., Sverzellati N. (2018). Pulmonary quantitative CT imaging in focal and diffuse disease: current research and clinical applications. Br J Radiol.

[12] Cho Y.H., Seo J.B., Lee S.M., Choe J., Lee D., Kim N. (2018). Quantitative CT imaging in chronic obstructive pulmonary disease: review of current status and future challenges. J Korean Soc Radiol.

[13] Chaudhary M.F., Hoffman E.A., Guo J. (2023). Predicting severe chronic obstructive pulmonary disease exacerbations using quantitative CT: a retrospective model development and external validation study. Lancet Digit Health.

[14] Fernandes L., Fernandes Y., Mesquita A.M. (2016). Quantitative computed tomography imaging in chronic obstructive pulmonary disease. Lung India.

[15] Shahin Y., Alabed S., Alkhanfar D. (2022). Quantitative CT evaluation of small pulmonary vessels has functional and prognostic value in pulmonary hypertension. Radiology.

[16] Barbosa E.J.M. (2016). Quantitative imaging of chronic obstructive pulmonary disease—moving towards clinical application. J Thorac Dis.

[17] Tang X., Xu C., Zhou T., Qiang Y., Wu Y. (2024). Association of low attenuation area scores with pulmonary function and clinical prognosis in patients with chronic obstructive pulmonary disease. Open Life Sci.

[18] Rahaghi F.N., Ross J.C., Agarwal M. (2016). Pulmonary vascular morphology as an imaging biomarker in chronic thromboembolic pulmonary hypertension. Pulmon Circ.

[19] Cajigas H.R., Lavon B., Harmsen W. (2023). Quantitative CT measures of pulmonary vascular volume distribution in pulmonary hypertension associated with COPD: association with clinical characteristics and outcomes. Pulmon Circ.

[20] Matsuoka S., Yamashiro T., Matsushita S. (2014). Relationship between quantitative CT of pulmonary small vessels and pulmonary perfusion. AJR Am J Roentgenol.

[21] Helgeson S, Baig H, Nadrous T Quantitative CT imaging with vascular analysis in combined pulmonary fibrosis and emphysema. https://publications.ersnet.org/content/erj/64/suppl68/pa1651.

[22] Zeder K., Sassmann T., Foris V., Douschan P., Olschewski H., Kovacs G. (2024). Severe pulmonary hypertension in chronic obstructive pulmonary disease—from clinical perspective to histological evidence. Int J Cardiol Congenit Heart Dis.

[23] Raza A., Gayen S. (2025). The difference in survival between different phenotypes of patients with COPD and pulmonary hypertension (COPD-PH). Am J Respir Crit Care Med.

[24] Tello K., Yogeswaran A., Majeed R.W. (2025). Association of phosphodiesterase-5 inhibitor treatment with improved survival in pulmonary hypertension associated with COPD in the pulmonary vascular research institute GoDeep meta-registry. Chest.

[25] Chaouat A., Bugnet A.S., Kadaoui N. (2005). Severe pulmonary hypertension and chronic obstructive pulmonary disease. Am J Respir Crit Care Med.

[26] Kovacs G., Agusti A., Barberà J.A. (2018). Pulmonary vascular involvement in chronic obstructive pulmonary disease. Is there a pulmonary vascular phenotype?. Am J Respir Crit Care Med.

[27] Blanco I., Tura-Ceide O., Peinado V.I., Barberà J.A. (2020). Updated perspectives on pulmonary hypertension in COPD. Int J COPD.

[28] Olsson K.M., Hoeper M.M., Pausch C. (2021). Pulmonary vascular resistance predicts mortality in patients with pulmonary hypertension associated with interstitial lung disease: results from the COMPERA Registry. Eur Respir J.

[29] Zeder K., Sassmann T., Foris V., Douschan P., Olschewski H., Kovacs G. (2024). Severe pulmonary hypertension in chronic obstructive pulmonary disease—from clinical perspective to histological evidence. Int J Cardiol Congenit Heart Dis.

[30] Barberà J.A., Peinado V.I., Blanco I. (2024). Untangling severe pulmonary hypertension in chronic obstructive pulmonary disease. J Heart Lung Transplant.

[31] Chaouat A., Naeije R., Weitzenblum E. (2008). Pulmonary hypertension in COPD. Eur Respir J.

[32] Rivera Robles J.D., Ahmad I., Pendlebury D. (2023). COPD Phenotypes: Looking Outside the Lung.

[33] Carlsen J., Andersen K.H., Boesgaard S., Iversen M., Steinbrüchel D., Andersen C.B. (2013). Pulmonary arterial lesions in explanted lungs after transplantation correlate with severity of pulmonary hypertension in chronic obstructive pulmonary disease. J Heart Lung Transplant.

[34] Bunel V., Guyard A., Dauriat G. (2019). Pulmonary arterial histologic lesions in patients with COPD with severe pulmonary hypertension. Chest.

[35] Elbehairy A.F., Vincent S.G., Phillips D.B. (2023). Pulmonary vascular volume by quantitative CT in dyspneic smokers with minor emphysema. COPD.

